# Role of Electrical Impedance Tomography in Clinical Practice in Pediatric Respiratory Medicine

**DOI:** 10.1155/2013/529038

**Published:** 2013-12-25

**Authors:** Wojciech Durlak, Przemko Kwinta

**Affiliations:** Department of Pediatrics, Jagiellonian University Medical College, ul. Wielicka 265, 30-663 Krakow, Poland

## Abstract

This paper summarizes current knowledge about electrical impedance tomography (EIT) and its present and possible applications in clinical practice in pediatric respiratory medicine. EIT is a relatively new technique based on real-time monitoring of bioimpedance. Its possible application in clinical practice related to ventilation and perfusion monitoring in children has gaine increasing attention in recent years. Most of the currently published data is based on studies performed on small and heterogenous groups of patients. Thus the results need to be corroborated in future well-designed clinical trials. Firstly a short theoretical overview summarizing physical principles and main advantages and disadvantages is provided. It is followed by a review of the current data regarding EIT application in ventilation distribution monitoring in healthy individuals. Finally the most important studies utilizing EIT in ventilation and perfusion monitoring in critically ill newborns and children are outlined.

## 1. Introduction

Electrical impedance tomography (EIT) is a relatively new, noninvasive monitoring technique. Its usefulness in lung function monitoring in adults has been examined in numerous studies. The increasing number of publications in pediatric population in recent years shows a wide range of clinical applications of this method. Unlike conventional radiography or computed tomography (CT), EIT is radiation-free and enables continuous real-time monitoring of the lung function. The current knowledge about the application of EIT in clinical practice in children will be reviewed in this paper.

## 2. Physical Principles

The theoretical aspects of electrical impedance tomography were described over 30 years ago [1]. The physical principle is based on the measurement of intrathoracic bioimpedance distribution. Standard electrodes are placed circumferentially on the surface of the patient's chest. High-frequency, low-amplitude electrical current is applied sequentially to the chest by the consecutive pairs of electrodes. The surface potential is measured by the remaining pairs of electrodes. Most currently available systems are equipped with 16 electrodes. One complete rotation creates a voltage profile, or a frame which is used for reconstruction of a cross-sectional tomographic image of the chest. The reconstruction is based on the Sheffield backprojection reconstruction algorithm. The picture represents distribution of impedance in different regions of the lungs. However this provides very little information about changes of impedance in time. Following the first reports, in 1995 the idea of functional EIT (fEIT) monitoring was devised [2]. This modality allows visualizing of local and global impedance changes over specific period of time creating a dynamic map of ventilation distribution. The results are presented as color-coded cross-sectional images of the chest. The regions with high levels of impedance changes (Δ*Z*) represent well-ventilated segments; whereas low Δ*Z* corresponds with low regional ventilation. Taking the concept one step further, the idea of ΔfEIT [3] monitoring was introduced. The aim of this method is subtracting two fEIT pictures obtained at different times in different clinical circumstances and establishing the difference between them.

The precise quantification of absolute impedance values is challenging and requires taking many factors into account, including specific information about the shape of the chest. Thus EIT is more suitable for assessing dynamic impedance changes in time. Substantial effort has been made in order to quantify information obtained during EIT monitoring, which resulted in several numerical indices of EIT results that have been described in detail in a recent review [4].

EIT has been extensively validated as a reliable ventilation distribution monitoring technique in both healthy and lung-injured animal models, as well as in humans. Validation data confirms that EIT is a highly reproducible method and that impedance changes reflect changes in regional ventilation accurately [5–9].

Electrical impedance tomography has several potential disadvantages. Most importantly, the relatively low spatial resolution precludes using it to obtain morphological information, as computed tomography and magnetic resonance both offer substantially higher resolution. EIT is also more suitable for observing dynamic changes in time in one particular patient, rather than for interindividual comparison. The other technical issue is associated with feasibility of electrodes placement, especially in extremely prematurely born children.

## 3. Ventilation Monitoring in Healthy Children

The pulmonary function testing in children, neonates, and infants in particular poses considerable technical difficulties due to the inability of these patients to cooperate. EIT offers a promising alternative tool that can be used to monitor regional ventilation distribution over extended periods of time, with no need of sedation or cooperation of the patient.

Brown et al. examined the dynamics of total lung resistivity in the first months of life in healthy newborns using EIT [10]. They conclude that lung resistivity increases over the first 3 years of life with the most significant increase in the first 3 months reflecting rapid maturational changes in the lung structure. They are considered to be related to extracapillary blood volume changes and alveolar wall structure changes rather than the increasing number of alveoli.

The body position is known to change ventilation distribution in both adults and children. According to the long-existing paradigm, based on studies performed over 20 years ago [11, 12], nondependent lung areas in infants are better ventilated than dependent ones, opposite to the pattern observed in adults [13].

The study by Frerichs et al. [14] reported that EIT provides regional functional data not otherwise obtainable at the bedside. The authors emphasize the high variability of the regional ventilation depending on the body position and breathing pattern in a small group of healthy newborns. In addition they report a high irregularity in breathing patterns in all monitored patients. A similar study was published by Heinrich et al. in 2006 [15]. The authors compared the regional lung ventilation using EIT in spontaneously breathing infants and intubated, mechanically ventilated ones. They conclude that left lung tidal volumes are decreased in the supine position with the head turned to the left as well as in the prone position, regardless of the head position in the spontaneously breathing patients. The tidal volumes in the left lung of the mechanically ventilated children were only affected in the prone position with the head turned leftwards. However, both of these studies are based on very small groups of patients, which precludes drawing any solid conclusions.

A study published by Schibler et al. in 2009 [16] defies the existing view of the reversal model of regional ventilation distribution. The authors conclude that in the group they studied the ventilation was distributed equally between dependent and nondependent lung-regions and the pattern observed in neonates is not dissimilar to that observed in adults.

Another study published by the same group [17] evaluated ventilation distribution in spontaneously breathing healthy newborns in the supine position, with a 6-month follow-up. According to the authors the posterior (dependent) aspects of the lungs were better ventilated than anterior (nondependent) aspects throughout the follow-up time. There is also a more significant increase in the posterior ventilation comparing to the anterior area with age. They also stated that the posterior regions of the lungs increase their volume before anterior during tidal ventilation in supine position. The results of the study that was recently published [18] also oppose the pattern that was accepted previously and emphasize the complexity of the ventilation distribution in healthy nonsedated infants and children. The authors report that only 35% of participants showed greater ventilation of the nondependent regions, whereas 15% consistently showed an opposite pattern and most importantly in a group of 51% a variable pattern was observed. These opposing results need to be addressed and clarified in further studies performed on larger groups of patients.

## 4. The Use of EIT in Ventilation Monitoring in Critically Ill Newborns and Infants

In recent years, a growing number of studies try to address the problem of feasibility and usefulness of EIT as a noninvasive monitoring method in newborns and infants with different respiratory and other pathologies. Those studies widely contribute to gaining more extensive knowledge about respiratory pathophysiology.

The study published in 2001 by Frerichs et al. [19] was one of the first assessing applicability and clinical relevance of EIT in neonatal and pediatric intensive care unit (ICU) setting. In this study a small group of patients with miscellaneous respiratory conditions were monitored using EIT. Based on the results, authors suggest that EIT might be beneficial for the patients in ICU in monitoring regional lung ventilation during surfactant administration, which could diminish prevalence of complications associated with volutrauma. They also infer that the adjustment of ventilatory settings could be guided by the impedance signal changes. The third aspect of possible EIT application that is discussed in the article is monitoring of gravity-related ventilation distribution shifts. This issue seems to be particularly important since the body position is frequently changed in critically ill infants in order to modify the breathing mechanics. The authors conclude that these aspects need to be addressed in further studies on more homogenous groups of patients.

Several studies were aimed at establishing the influence of different therapeutic procedures on regional ventilation distribution in critically ill children. The paper published by Wolf et al. in 2007 [20] assessed the magnitude of tidal volume changes during airway suctioning in a small group of mechanically ventilated children diagnosed with acute respiratory distress syndrome (ARDS). The authors demonstrate regional lung volume differences during mechanical ventilation and derecruitment maneuver (closed-line airway suctioning). The magnitude of tidal volume loss measured with EIT was reported to be more significant in nondependent lung regions. Similar study by van Veenendaal et al. [21] assessed the influence of closed endotracheal tube (ETT) suctioning on lung volume in prematurely born children with RDS treated with high-frequency ventilation. They were able to show in a group of 12 newborns that EIT is a reliable method for assessing lung volume changes during and after ETT suctioning. The study shows sudden and steep drop in lung volume reflected by decrease of global impedance in all children enrolled.

Surfactant treatment efficacy in prematurely born children with respiratory distress syndrome (RDS) poses a very promising target for application of EIT as a monitoring tool. Until recently, the effectiveness of surfactant treatment was assessed based on patient's clinical improvement, with very little insight into direct effect of surfactant on regional and global ventilation and respiratory mechanics. This issue was addressed in a paper by Miedema et al. [22]. The authors used EIT to monitor prematurely born children with RDS treated with high frequency ventilation (HFV) and with surfactant and were able to prove that surfactant causes rapid increase in lung volume with subsequent stabilization and increased lung compliance. In another recently published study [23], the authors used EIT to assess influence of surfactant on regional and global lung ventilation in a group of 17 newborns with RDS treated with conventional mechanical ventilation. They report that surfactant treatment increases lung resistivity and normalized impedance change (Δ*Z*) reflecting increase of lung compliance and ventilation, respectively.

One of the most important aspects of EIT application in clinical practice is describing the regional inhomogeneity in lung ventilation. Even though pneumothorax seems to be a plausible condition in this context, there is only one casuistic publication that reported using impedance monitoring in a newborn with pneumothorax [24]. Miedema et al. report a significant asymmetry of tidal and oscillatory ventilation, favoring the unaffected left side. However, due to lack of EIT data prior to onset of pneumothorax and after thoracic drain placement, this report poses significant limitations.

EIT-guided ventilatory support is the major hope and ultimate goal associated with this modality. Adequate ventilation settings adjustment is crucial for minimizing the risk of volutrauma and barotraumas but ensures sufficient ventilation on the other hand. It has become clear that global parameters such as arterial blood gases or lung compliance are insufficient in ventilation efficacy and safety monitoring. Recently published paper [25] proves that EIT is a reliable tool to assess the influence of continuous positive airway pressure (CPAP) level on end-expiratory lung volume (EELV) in preterm newborns with RDS. Moreover it shows a homogenous increase of lung volume with increasing level of CPAP in the range of 2 to 6 cm. Results of a small feasibility study [26] that was published in 2013 corroborate this data. The study was aimed at assessing whether EIT is a suitable method for determining optimal positive end-expiratory pressure (PEEP) level that provides the most homogenous ventilation in mechanically ventilated very low birth weight (VLBW) infants prior to extubation. In conclusion the authors emphasize that the level of PEEP providing optimal homogeneity of ventilation could be higher than routinely used in clinical practice. These results suggest future application of EIT in functional assessment of the respiratory system in children before extubation. Animal model studies also confirm that EIT-guided ventilation might be beneficial and decrease frequency of ventilatory-associated trauma [27].

Another possible EIT application that was recently described is the endotracheal tube position assessment [28]. Based on this small feasibility study, EIT might be considered a reliable tool for detecting the correct as well as incorrect positioning of the ETT (including both main bronchus and oesophagus). This was confirmed on an animal model in a study by Schmölzer et al. [29]. The authors compared EIT with five other noninvasive techniques concluding that EIT might be a reliable method, however, emphasizing that none of the investigated methods were without limitations and that the combination of all of them should be used in clinical practice.

## 5. Perfusion Monitoring

Significant efforts have been made to assess the feasibility of EIT-based lung perfusion monitoring, as this could improve our understanding of ventilation-perfusion mismatch and its role in respiratory pathophysiology. The current state of knowledge about this issue was summarized in recently published reviews [4, 30]. We found only one clinical study addressing this problem in pediatric population [31]. This paper emphasizes the feasibility of perfusion monitoring using EIT in infants. The authors were able to show that the amplitude of impedance changes measured in the circulatory frequency domain was higher in the anterior part of the chest than in the posterior part in the supine infants. This might reflect the impact of regional pulmonary perfusion changes; however it needs to be further evaluated in future studies.

## 6. Summary

Electrical impedance tomography is a very promising technique for noninvasive, radiationfree monitoring of lung function. It is widely applicable and safe for the patient, allowing real time continuous monitoring of mechanical properties of the lungs over extended periods of time. Even though the physical principle of EIT is thirty years old, we have seen increasing number of publications suggesting its possible application in clinical practice in the recent literature. This is mainly due to constantly improving quality of hardware and data processing modalities. However most of these studies were based on small heterogeneous groups of patients. Thus, larger, well-designed trials are necessary to assess the role of electrical impedance tomography in everyday clinical practice.
